# Benefits of EPAs at risk? The influence of the workplace environment on the uptake of EPAs in EPA-based curricula

**DOI:** 10.1007/s40037-021-00658-9

**Published:** 2021-03-31

**Authors:** Karsten Arthur van Loon, Linda Helena Anna Bonnie, Nynke van Dijk, Fedde Scheele

**Affiliations:** 1 School of Medical Sciences of the Amsterdam University Medical Center, Amsterdam, The Netherlands; 2 Department of General Practice of the Academic Medical Centre, Amsterdam, The Netherlands; 3 Health Systems Innovation and Education at the VU University in Amsterdam and Amsterdam UMC, OLVG, Amsterdam, The Netherlands

**Keywords:** Postgraduate medical education, Entrustable professional activities (EPAs), Curriculum development

## Abstract

**Introduction:**

Entrustable Professional Activities (EPAs) have been applied differently in many postgraduate medical education (PGME) programmes, but the reasons for and the consequences of this variation are not well known. Our objective was to investigate how the uptake of EPAs is influenced by the workplace environment and to what extent the benefits of working with EPAs are at risk when the uptake of EPAs is influenced. This knowledge can be used by curriculum developers who intend to apply EPAs in their curricula.

**Method:**

For this qualitative study, we selected four PGME programmes: General Practice, Clinical Geriatrics, Obstetrics & Gynaecology, and Radiology & Nuclear Medicine. A document analysis was performed on the national training plans, supported by the AMEE Guide for developing EPA-based curricula and relevant EPA-based literature. Interviews were undertaken with medical specialists who had specific involvement in the development of the curricula. Content analysis was employed and illuminated the possible reasons for variation in the uptake of EPAs.

**Results:**

An important part of the variation in the uptake of EPAs can be explained by environmental factors, such as patient population, the role of the physician in the health-care system, and the setup of local medical care institutions where the training programme takes place. The variation in uptake of EPAs is specifically reflected in the number and breadth of the EPAs, and in the way the entrustment decision is executed within the PGME programme.

**Discussion:**

Due to variation in uptake of EPAs, the opportunities for trainees to work independently during the training programme might be challenging. EPAs can be implemented in the curriculum of PGME programmes in a meaningful way, but only if the quality of an EPA is assessed, future users are involved in the development, and the key feature of EPAs (the entrustment decision) is retained.

**Supplementary Information:**

The online version of this article (10.1007/s40037-021-00658-9) contains supplementary material, which is available to authorized users.

## Introduction

Across the world, programmes for postgraduate medical education (PGME) use competence frameworks, such as the Canadian Medical Education Directives for Specialists (CanMEDS) or the framework of the Accreditation Council for Graduate Medical Education (ACGME), as the foundation of their training. These frameworks describe the competences that health-care professionals should acquire during their training [1] and are used for assessment and for structuring the trainee learning process [2]. Competences are seen as descriptors of a physician’s personal quality. However, in the workplace, where most learning by health-care professionals takes place, using competences for assessment purposes appears to be difficult. Both trainers and trainees have indicated that competence frameworks are too abstract and theoretical to be applied in workplace daily practice [2, 3]. Additionally, most assessment tools only focus on a limited number of competences. As a result, the overall aim of the competence frameworks, which was to ensure a comprehensive approach to medical education, is under threat [4, 5].

To support the use of competence frameworks in PGME programmes without compromising the comprehensive approach, Ten Cate et al. developed the concept of entrustable professional activities (EPAs) [2, 3, 6, 7]. EPAs are ‘units of professional practice, defined as tasks or responsibilities to be entrusted to unsupervised execution by a trainee once he or she has attained sufficient competence’ [2]. EPAs combine the assessment of competences described in a competence framework with the activities that must be performed in the daily practice of a medical specialty [3, 6]. When applying EPAs for the assessment of competences in daily clinical practice, their key feature is the entrustment decision: can the trainee be trusted to bear responsibility, and is the trainee, consequently, allowed to perform the professional activity independently [3, 6–8]? In theory, when a trainee has demonstrated sufficient proficiency in the competences specified in an EPA, a trainer can decide to fully entrust a trainee to perform the activity independently and with supervision at a distance [3, 7]. In this way, EPAs can be used to support assessment in a competence-based training programme.

EPAs can also be used as building blocks when constructing a curriculum. Starting with a description of EPAs, rather than competences to be achieved, helps curriculum developers to articulate important holistic activities in which trainees should be educated [3, 7]. A description of the EPAs also provides opportunities for EPAs to shape trainees’ learning processes. This takes place not only by means of a rich description of training goals, but also by means of feedback provided by a trainer on trainee performance in an EPA [3, 7].

Currently, EPAs have been introduced into various PGME programmes. In the Netherlands, where this study was situated, changes in national legislation led to a need for trainee-tailored, competence-based training programmes, which resulted in the development of EPA-based curricula in Dutch PGME programmes [9–11]. Extensive guidance in developing EPAs and EPA-based curricula is provided by an AMEE Guide [7] and by the Dutch Association of Medical Specialists [11]. However, the uptake of EPAs in the curricula varies among the different specialty training programmes [12–19]. The reasons for and consequences of this variation remain unknown but are of interest for developers of PGME programmes in helping them make well informed choices when introducing EPAs in their curricula.

The unique aspects of the workplace environment of a training programme may be one of the factors that leads to decisions about the uptake of EPAs in a curriculum that does not completely align with the original aims behind the use of EPAs [3, 20, 21]. The current literature provides little insight into how these training programmes might diverge from the intended use of EPAs as described by the founder, Olle ten Cate. It is also unknown whether the benefits of working with EPAs are at risk if the method is reinterpreted in light of the specific environment. Therefore, our main research question was: ‘How does the workplace environment of various PGME programmes influence the uptake of EPAs in their national training plans?’ We set out to answer this research question with a case study that evaluates how EPAs are applied in the national curricula of four PGME programmes with different workplace environments. Based on the research results of this study, we aimed to evaluate the extent to which the benefits of working with EPAs are at risk when the uptake of EPAs in national training plans is influenced by the workplace environment.

## Methods

### Setting

In the Netherlands, for economic reasons, the duration of many PGME programmes had to be reduced after 2014 [22]. To realize this goal, while maintaining the quality of the training programmes, trainees are now offered a tailored PGME programme, in which training duration depends on the trainee’s available knowledge and skills and how quickly a trainee acquires further competences. To implement this new educational approach, various Dutch PGME programmes applied the EPA method to facilitate tailored competence-based training [9, 10, 12, 14, 17, 18, 23]. The outlines of all Dutch PGME programmes are described in national curricula, taking into account national laws and regulations for PGME. Local training institutes use these national curricula as guidelines for the execution of the training programme.

For this study, we selected four Dutch PGME programmes that describe EPAs in their curricula, namely in the specialties of general practice, clinical geriatrics, obstetrics & gynaecology, and radiology & nuclear medicine. These four PGME programmes were selected because of their varying workplace environments, including intramural and extramural health care, different patient populations, and more specialized versus holistic care. (Tab. 1; [12, 14, 17, 18]).

**Table 1:** Variation in context of PGME programmes

Variation in working environment of PGME programmes				
*Medical Specialty*	*Feature of medical specialty*	*Location*	*Patient population*	*Patient contact*
General practice	General care with a holistic perspective	Extramural	High variety in patient population	Intensive direct patient contact
Clinical geriatrics	Specialist care with a holistic perspective	Intramural	Specific patient population	Intensive direct patient contact
Obstetrics & gynaecology	Specialist care	Intramural	Specific patient population	Intensive, direct patient contact
Radiology & nuclear medicine	Specialist care	Intramural	High variety in patient population	Little direct patient contact

### Study design

For this study an interpretivist approach was taken to understand and interpret the choices which were made in the curricula. The first part of this qualitative case study consisted of a document analysis. KvL and LB requested the scientific boards of the four PGME programmes, who are responsible for creating curriculum plans, to send the latest version of the national curriculum plan for their programme. The plans were analysed to determine how the EPAs were incorporated into the curricula of the PGME programmes.

The second part of this study consisted of semi-structured interviews, held between June 2017 and August 2017. KvL and LB, both with extensive interviewing experience, conducted the interviews, which were audio-recorded lasting a maximum of 45 min. All audio-recordings were transcribed verbatim and analysed using MAXQDA [24]. KvL and LB asked interviewees to explain their choices concerning how EPAs were incorporated into their curriculum. The interview guide (see Appendix 1 in the Electronic Supplementary Material) was based on the document analysis and AMEE Guide for the development of an EPA-based curriculum [7]. This study was approved by the Ethical Review Board of the Netherlands Association for Medical Education (NVMO) (ERB file no.: 871).

### Participants

KvL and LB invited, via email, four medical specialists to be interviewed, one from each of the four selected PGME programmes. We selected these interviewees based on their active involvement in the development of the EPA-based curriculum for their medical specialty. In addition, we selected interviewees who were involved in implementing the national curricula in practice, so all participants were able to reflect on the consequences of the uptake of EPAs on training practice. All four interviewees responded positively to the invitation and appointments were made for face-to-face interviews in their workplaces. Researchers did not have any relation with the interviewees and were not involved in the daily work of the interviewees.

### Data analysis

#### Document analysis

KvL and LB analysed two curriculum plans each and cross-checked each other. Aligned with document analysis, we combined elements of content analysis and thematic analysis [25]. The analysis of content was first done by finding, selecting, appraising, and synthesising data from the curriculum plans. This process helped in selecting the paragraphs describing the uptake of EPAs in the training programmes. Next, KvL and LB made a selection of themes concerning the most relevant applications of EPAs (Tab. 2), based on the above mentioned AMEE Guide [7] and relevant literature on EPA use [20, 26, 27]. After discussion between all authors, three themes were selected with the main elements of EPAs, as described in the studies introducing EPAs in medical education, in mind [3, 6]. Finally, KvL and LB used the themes for the thematic analysis of the curriculum plans.

**Table 2:** Possible EPA applications

Possible areas of application	Description
Assessment of competences and entrustment decision for independent practice	In EPAs, the competences are mapped onto activities from daily practice. In order to be able to perform an EPA, multiple competences from several domains have to be applied at the same time. Assessment of competences takes place by means of determining how much supervision the trainee needs for the specific EPA. An EPA can be entrusted to a trainee once he/she has demonstrated the competences of the EPA concerned and once he/she is able to manage the risks that are involved in the EPA under all circumstances. By gradually increasing the level of entrustment to the trainee, the level of responsibility and autonomy of the trainee can also gradually increase because of a decrease in the level of supervision
Professional development	Using the EPAs, an individual curriculum can be built, indicating the moments at which the levels of supervision are expected to be reached by the trainee. This individual curriculum can guide both trainee and trainer in learning. Knowing what level of supervision is expected at which moment, the trainee and the trainer are both given directions for learning and teaching. The addition of elective EPAs may add focus to areas of interest of the trainee, thereby further individualizing the personal development plan of the trainee
Curriculum development	A workplace curriculum can be built using a set of validated EPAs

#### Interviews analysis

We used conventional content analysis to gain insight into the uptake of EPAs in Dutch PGME programmes [28, 29]. Since existing theory or literature on the uptake of EPAs is limited, no preconceived themes were used [19]. KvL and LB co-analysed the transcripts. After intensively reading the four transcripts, we applied an inductive coding process to all four transcripts. Subsequently, we created a codebook to identify all the emerging themes regarding the uptake of EPAs. All authors extensively discussed the themes and compared them to the results of the thematic analysis of the document analysis to bring out the various ways in which EPAs were formulated in PGME programmes.

All researchers had experience in creating curriculum plans. One of them (FS) was actively involved in the uptake of EPAs in obstetrics and gynaecology training. Triangulation between researchers was reached with researchers from both medical and educational backgrounds, a varying level of experience and training in analysing data, and acquaintance with both in- and out-hospital training programmes.

## Results

The document analysis and the interviews with curriculum developers gave insight into the choices which were made concerning the uptake of EPAs in the national curricula. In all four examined training programmes, EPAs were the main building blocks of the national curriculum. All training programmes aimed to contextualize competencies with help of EPAs to specify learning goals that were recognizable for both trainers and trainees. Differences in the uptake between the four programmes were found in three different themes: curriculum development, professional development, and assessment.

### Curriculum development

Regarding the use of EPAs for curriculum development, the document analysis showed that the number of EPAs differed between the four training programmes varying from 12 to 120 EPAs per programme. Interviewees stated that the number of EPAs in the curriculum depended on the training context of the PGME programmes. The four training programmes differ greatly with regard to patient population, the work that has to be done, and the clinics in which patients are treated (Tab. 1). These differences influenced the uptake of EPAs for curriculum building. For general, more holistically oriented practices, EPAs needed to be formulated more generally. More specialized training programmes with many procedures can handle more narrowly framed EPAs, since procedures are easier to observe than holistic activities.

*And our philosophy of individual training and competence-based training is that, if someone is able to perform in a specific sub-area, (…) then he or she does not always need supervision for that. And that was the trigger for us to say; we shouldn’t work with those big EPA blocks, because otherwise we can’t give a trainee independence when he or she could have independence.*

—Radiology

### Professional development

EPAs were used for professional development of trainees. EPAs provided a clear overview of the skills and competencies that trainees have to master during the PGME programmes; EPAs provided guidance for trainers in coaching trainees and in giving trainees feedback; and EPAs provided guidance for trainees in formulating a personal development plan and setting learning goals for the upcoming training period. All four analysed training programmes described EPAs as a tool for individual professional development.

*They [EPAs] are now positioned to guide the trainees’ individual training plan […] So the trainee knows, when I start the training programme and I start working in GP practice, these are the themes that I have to focus on and these are the EPAs that I have to master.*

—General practice

The training programs that are more focused on specialty training explicitly described that the use of EPAs offer differentiation opportunities for trainees at the end of the training programme. For instance, in the training programme of radiology & nuclear medicine, trainees who are at the end of their training programme can choose to specifically focus on radiology or nuclear medicine. By contrast, the more holistically oriented programmes (GP and clinical geriatrics) instead used EPAs to determine what a trainee has to focus on to fulfil the final, relatively fixed requirements of training.

### Assessment

The way EPAs were intended to be used for assessment of trainees differed greatly between the four PGME programmes and varied from not using EPAs as an assessment instrument at all to assessing each individual EPA. Assessment with EPAs was done to determine the level of supervision a trainee needs. The three in-hospital training plans described EPAs as a tool for granting entrustment, which should make trainees independent of supervision. However, from all four national curricula plans it was unclear what practical consequences entrustment of an EPA had for the independent practice of trainees.

The small number of EPAs in the clinical geriatrics training made it difficult to give independence to trainees based on the level of entrustment. The obstetrics & gynaecology and radiology training have divided their curricula into many more EPAs, which makes it easier to assess one EPA and make residents accountable for that activity. Although this accountability is not described specifically in the curricula, interviewees stated it was the intention to use EPAs for making residents accountable during training.

*It at least provides tools to make [things] clear within your team: [if] someone can do it, it’s time to give him/her that trust.*

—Obstetrics & gynaecology

General practice training decided not to use EPAs for assessment, for fear of creating yet another checklist without consequences for independent practice. This means EPAs lost their meaning as units of assessment within this training programme.

*We have had a tough discussion: are EPAs meant to assess trainees? It caused a lot of resistance. That resistance consisted in the fact that people were afraid that a bureaucratic instrument would arise, in which trainees were not stimulated in their learning but stimulated to use another checklist.*

—General practice

The context in which the training takes place was of influence in how EPAs were used. GP training is characterized by its holistic role in patient care and its broad variation of patients which made it difficult to develop smaller, assessable EPAs. Even though clinical geriatrics cope with comparable holistic care they did aim for using EPAs for assessment.

*[…] in fact, our original attitude is that you only master the whole process at the end of the training programme, so we had to make concessions.*

—Clinical geriatrics

## Discussion

This study aimed to evaluate how the uptake of EPAs in the national training plans of various PGME programmes is influenced by the workplace environment, investigating whether the benefits of working with EPAs remain when they are adjusted to this environment. Our findings indicate that the uptake of EPAs differs across the various PGME programmes, with adjustments made so that EPAs can be used in the respective training programmes in a meaningful way. The variation in uptake is specifically reflected in the number and breadth of the EPAs, and in the way entrustment decisions are executed. The findings revealed that part of this variation in uptake can be explained by environmental factors related to the medical specialty of the PGME programme, such as patient population, the role of the physician in the health-care system, or the setup of local medical care institutions where the training programme takes place.

Previous research has shown that too much rigidity in the application of educational methods creates difficulties for those who must use them in the workplace [30, 31]. As a result, PGME programmes aiming to implement a new educational method such as EPAs have to make choices about the uptake of the new method in their curriculum in the light of the reality of daily practice within their own clinical environment [32–34]. Room for the reinvention of the concept and for diversity should be embraced since this can make it suitable for dissemination in daily practice [35]. However, it must be asked whether reinvention is still opportune if it is at the expense of the essential traits of a concept.

This returns us to the aim of this research to investigate if the benefits of working with EPAs are retained after their adaptation to specific work environments. In the GP training programme, EPAs lost their meaning with respect to assessing the independent practice of trainees, as the entrustment decision on independence was not a permanent element in the curriculum of the training programme. As the key feature of working with EPAs is being able to entrust trainees with independent practice during the course of the training programme [3, 6–8], it could be argued that the value of implementing EPAs is limited without this characteristic.

Furthermore, variation in the uptake of EPAs in the curricula of the PGME programmes was reflected in differences in the number and breadth of the EPAs described in the curriculum. As an entrustment decision should entail a significant step in the development, and in the independent practice, of a trainee, it is recommended in the literature that a training programme should have around 20–30 EPAs for a formal entrustment decision, with a maximum of 10 for every year of the training programme [7, 36]. The number of EPAs described was not only challenging for the PGME programmes in our study but has also proved to be an obstacle in other training programmes [37–40].

Previous studies have also shown that the adequate formulation of EPAs is challenging [41–44]. It has been acknowledged that a high number of EPAs, or EPAs that are overly abstract in their formulation, can have a negative impact on the assessment potential of the EPA. A high number of EPAs risks them becoming a checklist, without consequences for independent practice, while abstract EPAs might not provide sufficient support for trainers or trainees in working towards independent practice [43]. As a result, the benefit of EPAs leading to progressive independence in practice is at risk, which could mean that another critical benefit of working with EPAs is lost.

Thus far, a few tools have been created to support the development and implementation of EPA-based curricula, with the purpose of ensuring the meaningful use of EPAs in the workplace. For example, quality rating tools for EPAs, such as QUEPA [44] or Equal [42], combined with the AMEE Guide [7], can support training programmes in the development of high-quality EPAs.

Ultimately, however, the introduction of a new educational method into the curriculum of a PGME programme should not be focused on adhering to guidelines, but on empowering its future users (in the case of EPAs, the trainees or students and their trainers). Without the people who actually use the educational method in daily practice, the benefits of any educational method will never be reflected in practice. It is therefore important to gain insight into the opinions of future users about the new educational method at the beginning of the design of the new curriculum [45]. This process should be independent of the influence of the workplace environment on the educational method.

### Strengths and limitations

With the development of EPAs, Ten Cate et al. created an integrated educational method for workplace-based learning [2, 3, 6, 7]. This article took the opportunity to study and reflect on the highly varied use of EPAs in the country where the concept was developed, the Netherlands. Since the curricula have recently been renewed and the innovations have not yet been fully implemented in practice, we chose to focus on the uptake of EPAs in the national training plans of PGME programmes. However, in the meantime most training programmes have implemented EPAs and do have some experience with the method. Research into these experiences can be an addition for those considering or rewriting their EPAs. Moreover, we also focus on four Dutch PGME programmes. The study of other international programmes or specialties may furnish additional knowledge concerning the uptake and application of EPAs in PGME programmes. Additionally, only four medical specialists who were involved in the creation of the curricula were interviewed—one representative of each training programme. Although we were able to gain in-depth insights, we recognize that a larger sample size might expand our understanding.

### Future research

As we focused on EPA uptake in national training plans, we cannot draw conclusions about how EPAs are actually applied in the workplace. However, it is likely that with implementation in practice even more variation will arise. Future research could focus on differences between national curricula, and whether these differences are also found in the workplace. Moreover, it would be interesting to know whether the implementation of EPAs in national curricula leads to other forms of assessment, accountability, and professional development in practice. This would clarify whether EPAs create new forms of assessment that allow for progressive independence, as intended, or if EPAs merely replace our traditional assessment methods.

In conclusion, a variety of Dutch PGME programmes use EPAs in their national training plans. This study found that the uptake of EPAs in these training plans differs between programmes. These differences in uptake were a result of factors related to the workplace environment in which training in the medical specialties takes place. The variation in uptake of EPAs should not be considered a problem as long as the key feature of EPAs—entrusting trainees with independent practice during the course of the training programme—is honoured. However, a challenge may be that environmental factors can influence the number and breadth of EPAs, which might put opportunities for trainees to work independently during the course of a training programme at risk. Training programmes that aim to implement EPAs in their curricula should carefully consider specific environmental factors when developing EPAs, with the aim of ensuring their key feature is retained.

## Supplementary Information

Appendix 1 Interview guide
