# Supplementary material for: Benefits of EPAs at risk? The influence of the workplace environment on the uptake of EPAs in EPA-based curricula
Source: Perspect Med Educ. 2021 Mar 31;10(4):200–6. doi: 10.1007/s40037-021-00658-9 (PMC8368760; doi:10.1007/s40037-021-00658-9)
Supplement: Supplementary file 1 — Appendix 1 Interview guide [file 40037_2021_658_MOESM1_ESM.docx]

**Appendix 1** Interview guide

| Interviews are semi-structured. Besides the questions below, other questions can be added during the interview if relevant according to the interviewer. Before each interview, an informed consent statement is signed by both the interviewer and the interviewee. Interviews are audio recorded for data analysis only. Interviews have a duration of approximately 45 minutes.  *Background questions*   1. How are you involved in the post-graduate medical training of trainees? 2. In what manner were you involved in the creation of the national curriculum? 3. What is previous experience do you have designing curricula?   *Main questions*   1. How are, according to you, EPAs embedded in the national curriculum of your training programme? 2. What is the primary goal of EPAs in the national curriculum? 3. Are there any additional goals for using EPAs in the national curriculum? 4. What is the relationship between competencies and EPAs according to you? 5. In what way does the introduction of EPAs in your curriculum differ from the original EPA method? 6. What were the reasons for choosing a different approach? 7. In your view, does the way EPAs are being introduced in your curriculum support the assessment and independence of trainees? 8. Which organizational changes are needed to successfully implement EPAs in daily practice? |
| --- |
